# Differences in echocardiography interpretation techniques among trainees and expert readers

**DOI:** 10.1007/s12574-021-00531-y

**Published:** 2021-05-29

**Authors:** David Roy Anderson, Sarah Blissett, Patricia O’Sullivan, Atif Qasim

**Affiliations:** 1 505 Parnassus Ave. M1182, Box 0124, San Francisco, CA 94143 USA; 2 London Health Sciences Centre, 339 Windermere Rd B6 177A, London, ON N6A 5A5 Canada; 3 533 Parnassus Ave. Suite U-80, Box 0710, San Francisco, CA 94143 USA; 4 505 Parnassus Ave., Box 0214, San Francisco, CA 94143 USA

**Keywords:** Medical education, Fellowship, Trainees, Transthoracic echocardiogram (TTE), Adult echocardiogram, Concurrent think aloud (CTA) method, Qualitative case study

## Abstract

**Background:**

Trainees learn transthoracic echocardiogram (TTE) interpretation through independently completing and reviewing selected portions of the study with experts. The diagnostic accuracy of novice TTE interpretation is known to be low and schema for reading TTEs systematically are lacking. The purpose of our study is to identify techniques experts use while reading TTEs which could be used to more effectively teach novice readers.

**Methods:**

We performed a prospective qualitative case study to observe how experts and trainees interpret TTEs in an academic institution using a concurrent think aloud (CTA) method. Three TTEs of intermediate complexity were given to 3 advanced imaging fellows, 3 first year fellows and 3 expert TTE readers Participants filled out a report while reading and described aloud their thought processes. Sessions were video and audiotaped for analysis.

**Results:**

Experts and advanced fellows used specific techniques that novices did not including: previewing studies, reviewing multiple images simultaneously, having flexibility in image review order and disease coding, and saving hardest elements to code for the end. Direct observation of TTE reading informed trainee inefficiencies and was a well-received educational tool.

**Conclusions:**

In this single centered study we identified several unique approaches experts use to interpret TTEs which may be teachable to novices. Although limited in generalizability the findings of this study suggests that a more systematic approach to TTE interpretation, using techniques found in experts, might be of significant value for trainees. Further study is needed to evaluate teaching practices at other institutions and to assess whether implementation of these techniques by novices improves can improve their diagnostic accuracy and efficiency of reading at an earlier stage in their training.

## Introduction

The expanding clinical utilization of cardiac ultrasound, also known as transthoracic echocardiography (TTE) highlights the importance of training competent cardiologists in TTE interpretation [1–3]. However, unlike the teaching of ECGs there is no universally used systematic approach for trainees to learn to interpret transthoracic echocardiograms (TTEs) and diagnostic accuracy of novice TTE interpretation is low at 52% [4]. Before we can improve the diagnostic accuracy of fellows interpreting TTE, cardiologists must understand the factors contributing to this issue.

It is known that experts are more adept at recognizing patterns, organizing information for easier retrievability, and having a deeper understanding of the subject matter [7]. They may have more systematic approaches to reading which are not formally taught but have evolved through years of experience and review of many studies during their careers. Some of these may be transferable to novices to help them achieve proficiency earlier in their training.

In the field of radiology, studies have already identified techniques used by experts that may be transferrable to novices to improve their diagnostic accuracy. For example, radiology-based eye-tracking studies have identified differences in how novices and experts view still images, with experts and novices focusing on different features of the image [8–10]. After novices observed the eye-tracking patterns of experts interpreting images with lung nodules it was found that they had higher diagnostic accuracy in identifying lung nodules on CT [11]. While there are similarities between interpreting TTEs and diagnostic radiology images, there are differences including interpreting moving clips and, in some cases, interpreting more elements on an imaging study. Specific strategies of expert TTE readers may, therefore, differ from specific strategies used by expert radiologists.

The purpose of our study is to discover in detail how novice learners, intermediate learners, and experts interpret TTEs to identify and target interventions to improve trainee reading skills and efficiency.

## Methods

### Study design

We performed a prospective qualitative case study to observe how cardiologists and fellows interpret TTEs using a concurrent think aloud (CTA) method [12–14]. In the CTA method, individuals complete a task and speak out loud what comes to mind as they are performing it. CTA is a validated method to assess how individuals interact with a product or technology and is felt to reveal information about what is stored in a person’s working memory in the moment [13, 15, 16].

### Population and ethics

We recruited three cardiologists with greater than 10 years of experience reading echocardiograms (experts), three fellows at the end of their first year of cardiology training who had completed at least 3 months of echo rotations (novices) and three fellows in their final year of an advanced echocardiography fellowship (intermediates) to participate. All participants were either trainees or attending physicians at the University of California, San Francisco. All participants provided informed consent as volunteers without compensation. This study protocol was reviewed and approved by the IRB at UCSF (IRB number 16-19389).

### Materials

Three cases were initially chosen to have a representative sample of intermediate level TTEs. These TTEs were chosen after review and adjudication of the anonymized clips by two experts. We defined intermediate level echocardiograms as having a unifying diagnosis and one additional level of complexity such that an advanced echocardiography fellow (intermediate learner) would be expected to read each case correctly but with a reasonable level of difficulty. All cases had important findings for which a treating team would need to be notified in a real-world setting. The first echo case was 139 clips and highlighted a patient with carcinoid syndrome and severe tricuspid and pulmonary regurgitation. The second case, an 88 clip TTE study, was one of low gradient severe aortic stenosis in the setting of an ischemic cardiomyopathy. The third case, which was a limited study performed emergently with 57 clips, demonstrated a patient with a large pericardial effusion that was hemodynamically significant but also with concomitant pulmonary hypertension which masked some of the typical right sided features of tamponade.

### Procedures

All participants interpreted the three TTEs independently in the presence of an observer (AQ). The observer is an expert in TTE interpretation. A completely normal, pre-chemotherapy, practice TTE was included at the start of each session to help familiarize the participants to the CTA method.

The participant was asked to interpret the images and complete a report for each TTE. TTE images were viewed and reports were generated using Syngo (Version 10A, Siemens). Participants assigned a code (e.g., normal systolic function) to indicate their interpretations of the eight cardiac components in each report. A screen capture program (Snagit—TechSmith, Okemos, MI) was used for the computer monitors to record the process of how each reader scrolled through the specific echocardiogram images and videos, and to see how they manipulated the images or performed measurements. The observer asked the participant to describe their process of coding (the CTA method) and the order in which he/she was completing the task. Additional questions by the observer were used to augment the CTA method by clarifying statements made and to allow participants to better describe their thought process during the reading and coding.

After completion of this reading all participants were asked to reflect on the session for comments. Feedback was then provided by the observer to the trainees to improve their interpretation skills.

In addition to video and audio recording of the computer screen using Snagit, the entire process was also recorded using a digital audio recorder. Participants themselves were not videotaped. All audio from the observer (AQ) and participants was professionally transcribed.

### Data analysis

#### Quantitative analysis

The time taken for each reviewer to complete the echo report, from start to finish and including completing the TTE report, was recorded. Time for feedback at the end was not included in total time to review the TTE.

The generated TTE reports were analyzed for accuracy and compared against a gold standard of two experts (one from within UCSF and the other from the University of Pennsylvania).

Each TTE case was assigned a single unifying diagnosis: TTE 1 was severe tricuspid regurgitation in the setting of carcinoid disease, TTE 2 was low flow low gradient severe AS and TTE 3 was tamponade in the setting of severe pulmonary hypertension. In addition, each case was assigned 3 or 5 key elements felt to be essential parts of this single, unifying diagnosis. For TTE 1 there were 5 key elements: right ventricle function and tricuspid + pulmonary valve structure and function. For TTE 2 there were 3 key coding elements: LV function, regional wall motion abnormalities and severity of aortic stenosis. Finally, for TTE 3 there were 5 key coding elements: pulmonary artery pressure, effusion size, mitral or tricuspid Doppler respiratory variation, IVC size and right ventricle and atria collapse. Finally, a list of general coding elements felt to be essential for a comprehensive TTE report were selected by the adjudicating experts with TTE 1 having 21 elements, TTE 2 having 18 elements and TTE 3, a limited study, having 9 elements and is outlined in Table 3.

Each coded element (single diagnosis, 3 or 5 key elements and general coding elements) was designated as correct (1) or incorrect/missing (0) compared against a standardized report agreed upon by the adjudicating experts prior to study. The number of correctly codded key elements and number of correct total coded elements was recorded and is outlined in Table 1.

**Table 1:** Quantitative results from Cases 1–3 by level of training

**CASE 1**	Novices	Intermediates	Experts
Duration (average)	37 min	26 min	23 min
Coding Incorrect (% out of 21 total elements)	22%	13%	5%
Key Coding Elements Correct (5 total)	80%	86%	100%
Diagnosis Correct—Carcinoid	2/3	2/3	3/3

**CASE 2**	Novices	Intermediates	Experts
Duration (average)	26 min	26 min	23 min
Coding Incorrect (% out of 18 total elements)	19%	17%	10%
Key Coding Elements Correct (3 total)	78%	89%	100%
**Diagnosis Correct—Low Gradient AS**	0/3	2/3	3/3

**CASE 3** **﻿(limited study)**	Novices	Intermediates	Experts
Duration (average)	18 min	12 min	12 min
Coding Incorrect (% out of 9 total elements)	33%	30%	23%
Key Coding Elements Correct (5 total)	73%	73%	100%
Diagnosis Correct—Hemodynamically Significant Tamponade	2/3	2/3	3/3

Table outlines the quantitative results of novices fellows, intermediate or advanced imaging fellows and expert TTE readers for each of the three cases. Experts took less time to read each study with higher accuracy compared to other levels of learners. The perceived level of difficulty did not vary between training levels in all three cases

#### Qualitative analysis

Data from the CTA were extracted in multiple ways by two authors (DRA and AQ). First, the videotapes were reviewed to determine the order and method in which images were viewed and the sequence in which the elements on the report were filled out (which structures were commented and coded on first). After multiple reviews of these video and audio sessions key patterns, themes and characteristics were generated by each reviewer (DRA and AQ).

Using a qualitative content analysis approach [17], two authors (DRA and AQ) analyzed professionally transcribed documents for common words and phrases related to how each participant (novices, intermediates and experts) completed and coded the findings from the echocardiograms in Syngo. The observers then integrated and synthesized the findings from the audio and video recordings into ten characteristics that distinguish how novices, intermediates and experts interpret a TTE which direct quotes included in Table 2**.**

**Table 2:** Characteristics noted across different levels of expertise in echocardiogram reading

Characteristics	Novices	Intermediates	Experts	Representative Quotes
Number of Images viewed on screen at a tiime	One	Four	Four	Intermediate: “Often, it’s nice to read in four-view because you get… It’s a story, and you get more of the [overview]…each image isn’t isolated. So, I think it helps when you have four [images]. It tells the bigger story. You don’t get lost in the trees as much.”
Study Previewing before Reading/Coding	No	Yes	Yes	Intermediate: “I look through [the entire study] first because you’ve got to see what’s there, and also, you can really try and focus on the clinical picture. You kind of know your answer about the clinical question just going through once without writing anything on the template.”
“Chunking” or coding by specific categories	No	Yes	Yes	Expert: “I’m going to come back [to this part of the TTE] and put all those measurements into my report, but I just want to kind of get it kind of a gestalt of…of what’s going on [by previewing the whole study].” Novice: “I didn’t have a process or approach; this review has given me a new framework to approach reading TTEs.”
Re-measuring data	Yes	Yes	No	Novice: “Once again, not really liking the way they measured [this element]”
Reporting on Study Quality	No	No	Yes	Expert: “A little bit of a technically difficult study. Sort of not the best image quality, so you know, I might say fair.”
Focus of language used in the report to describe findings	No	No	Yes	Expert: “I think that is [wording] important because as you know…one of the things that I’m thinking about is what is the clinical implications of this patient.” Expert: “…the first thing I look at on this page, is she inpatient or outpatient, or where is she? And who ordered it? Because I’m thinking I might need to call them soon.”
Outside References Used to help interpretation	Yes	Yes	No	Novice: “I would look this up. This is an easy value to figure out.” “I’m going to pull out my phone just to calculate what the decrease is [across the mitral valve].” Novice: “There’s a website that I…a different website I use that has some valvular references.”
Coding the hardest elements at the end	No	Yes	Yes	Expert: “I’m going to come back [to this part of the TTE] and probably put all those measurements into my report, but I just want to kind of get it kind of a Gestalt of…of what’s going on [by previewing the whole study].”
Valued observation and feedback	Very helpful	Very helpful	Mildly helpful	Novice: There was never a standardized way. It was well, you sat with five different attendings for three minutes today and each one sort of talked out loud and got incorporated into the way that you [approach] an Echo Intermediate: “I found it really informative talking aloud and having the direct feedback on my reading style. This will really help my efficiency.”
Key “aha” moment slides that helped clinch diagnosis	No	Sometimes	Yes	Intermediate: “That’s pretty rare actually that you have the a-ha moment. In that case, um, I got hung up on the pulmonary valve. It wasn’t making sense, so the a-ha was when you saw the …you know, text book carcinoid picture.”

Table highlights ten characteristics that distinguish how novices, intermediates and experts interpret a TTE with direct quotes as examples. These results were obtained using a qualitative content analysis approach of professionally transcribed documents for common words and phrases related to how each participant (novices, intermediates and experts) completed and coded the findings from the echocardiograms in Syngo

## Results

### Time to interpret TTE and generate report

Novices took longer to read the 3 studies compared to intermediates and experts (Table 1). Experts typically read the studies fastest.

### Accuracy of TTE interpretation

Incorrect coding was more common among novice readers compared to the advanced fellow and expert groups (Table 1). In all the cases, experts documented and coded the correct final diagnosis, the most correct coded elements as well as all of the key elements for each case. Novice readers did not reliably report the final diagnosis (none were able to arrive at the correct diagnosis for case 2) despite reporting a high percentage of the general and key coded echocardiogram elements. One intermediate learner for each case (different individual for each case) missed the correct overall diagnosis, but overall did better than the novices.

### Characteristics that distinguish how fellows and experts interpreted TTEs

The ten characteristics that distinguished how the 3 expertise levels interpreted the TTEs are outlined in detail below and summarized in Table 2. Selected quotes highlighting examples of these characteristics are included.

1. Number of images viewed on screen
  All of the novices reviewed studies one still image or video clip at a time, whereas intermediates and experts used a **2 × 2 grid of 4** images at time and switched to a 1 image view when they needed to examine something more closely or perform a measurement. Novices were observed to have difficulty identifying the visualized anatomy when viewing 1 image at a time. For example, Novice 1 struggled with determining if he/she was looking at the right ventricular inflow view or right ventricular outflow view.
2. Study previewing before reading/coding
  Intermediates and experts often would **preview** the entire study first very quickly before filling anything out, whereas none of the novices did this. This was found to be most useful in the last case which was noted to be limited and unclear which images would be present.
3. “Chunking” or coding by specific categories
  Experts and intermediates would **aggregate information** based on the report template into 1 of the 8 categories found within the report template (left ventricular size and function, right ventricular size and function, atrial disease, valve disease, pericardial disease, aortic disease and pulmonary pressures, and miscellaneous). Novices attempted to code as many elements from each of these 8 categories as possible on the report when viewing each single image, whereas intermediates and experts would review multiple sets of images and video clips and **then fill out entire sections all at once**. As a result, novices frequently had to reenter or change their coding on certain element as they progressed through the case and gleaned new information (for example higher pulmonary artery pressures or valvular gradients).
4. Re-measuring data
  Experts were less likely to remeasure values to assess their accuracy when they didn’t make sense compared to a visual estimation, but rather decided whether the measurement was trustworthy compared to their eyeball test and then decided to keep it or reject it. This is contrast to the novices and the intermediates who were frequently observed re-calculating measurements when they did not trust them compared to visual estimation. For example, Novice 3 stated “once again, not really liking the way they measured [this element]” highlighting one of more than 5 times they re-measured gradients, areas, and volumes given by the echo technician.
5. Reporting on study quality
  All experts made it a point to **comment on study quality** to provide more context or qualification for their interpretations. Quality of study (graded as fair, good or excellent) was commented on by all experts and notably not mentioned by the novices or intermediates.
6. Focus on language used to the report to describe findings
  Furthermore, all experts used **distinctive wording** in their report to indicate urgency and provide clinical guidance for next steps or additional evaluation if needed. Moreover, they were more concerned about the nature of the language used to make sure it clearly outlined critical findings and clinical implications. Representative quotes from experts are outlined in Table 2 showing how they may use distinctive wording in their report and/or call the team for critical findings even before completing the TTE report.
7. Outside references used to help interpretation
  Experts did not use outside references for their reads, whereas novices and intermediates frequently used online resources such as Echocardiographer.org or the Calculate app by QxMD when making re-calculations and to answer questions that surfaced during reading.
8. Coding the hardest elements at the end
  Intermediates and experts were more flexible in their approach to completing the study report as they frequently **went out of order** and specifically made it a point to save the harder parts of the study for the end—for example assessing for wall motion abnormality, which requires review of multiple images from the beginning middle and end of the study.
9. Valued observation and feedback
  All trainees commented to the high value of directed feedback that was a byproduct of undertaking this study. All novices reported they had never been watched reading and filling out a report for a study from start to finish. They also felt having an observer watch them read a study without specific interruption or correction, followed by commentary and review of some of the reading techniques or methods as well as the final diagnosis, was very helpful in reducing wasteful practices and creating a more systematic approach to reading.
10. Key images/clips sometimes helped clinch a diagnosis

On one of the studies (carcinoid case) there was a one image (the RV inflow view) which for the experts and for some of the intermediate readers led to a moment, where the diagnosis was immediately certain. In this image the tricuspid valve leaflets were restricted and immobile, which is pathognomonic for this condition. Having seen prior examples of this type of condition led to the correct diagnosis immediately for the more experienced readers. None of the novices had such a moment.

## Discussion

This is the first study to describe the characteristics of three different levels of TTE readers in an academic setting. At our institution there were clear differences in how novice learners, intermediate learners, and experts interpret TTEs when it came to processing strategies (number of images reviewed at once, previewing studies, order in which images are viewed, order in which report template is completed, re-measuring values, using outside references, key clinching or “aha” moments), to the information reported (including study quality, language used to indicate critical findings) and to the value of being observed. Understanding the limitations that come with a small, single centered study we feel that some of these differences are hypothesis generating and may highlight how we can begin to develop a systematic approach to teaching trainees TTE interpretation.

Previewing the entire study first is a technique used by experts which might help novices with efficiency. For example, seeing whether contrast images are included at the end, whether there are additional views to assess maximal valve gradients can reduce unnecessary measurements or struggling with wall motion assessment during viewing of early images. The sequence in which experts viewed images and completed the report may also have advantages from a cognitive processing perspective. Chunking or sectioning information minimizes the strain on working memory by grouping like information together [18]. Because working memory is fixed, it is also felt that previewing the study and constructing an approach to reading each TTE, as we observed our experts do, is likely to free up mental processing and as a result reduce coding errors. This organized approach avoids the inefficiencies faced by novices who were observed coding elements based on the template, reviewing the same, single image multiple times and scanning back and forth through the entire study.

While intermediates and experts all viewed four images at a time, novices did not. Understanding that there are institutional differences in the way TTE is taught, based on the results in this study it is possible that reviewing one image at a time is perceived by the early learner as easier to process. However, one image viewing went hand in hand with novices trying to code multiple elements based on that single image and struggling to identifying structures without the context of other images. In our current training method experts review salient features of a pre-reviewed study one image at a time with novice learners which may be a reason we see early trainees read one image at a time. Teaching novices to begin to adopt a strategy, where four images are viewed at once may have distinct advantages later as they become more familiar with imaging protocols and can make scanning or previewing easier. This may also help address the fact that novices struggle to provide context to TTE images when viewed one at a time. For example, seeing multiple images including a Doppler across a valve structure next to the 2D and color images may help better identify the tricuspid valve versus the pulmonic valve in the parasternal images.

Another strategy that advanced readers employed was to code the hardest elements last. They filled out the easiest elements first and focused on items that would require the greatest amount of working memory last (i.e., assessing for wall motion abnormalities by reviewing multiple 2D images throughout the study at the end or focusing on valve disease severity after seeing all of the VTI data). This is an additional intervention that can be taught to help novices improve reading and may increase efficiency in filling out the less complex elements of the reports.

Experts included study quality and focused more on the language they use to describe findings in the final report to better communicate findings and provide a sense of urgency to the ordering physicians. The way the reports are worded can have an impact on downstream testing and clinical action and neither group of trainees focused or commented on the language they used in their report. Perhaps this is because they know it would be overread; however, this will eventually be an important professional obligation and it is worth considering teaching the appropriate language to use in the echo reports to avoid miscommunication. Addressing these gaps earlier in training may help with professional development.

Finally, an important finding that resulted from the feedback session was that none of the trainees had ever had someone directly observe them interpret images or fill out the report template for a TTE from start to finish. While this can take up to 30 min and may be challenging within the time constraints of an echo lab, providing real-time feedback on reading errors, efficiency, skills and techniques was highly valued from the trainee perspective. Direct observation in one randomized controlled trial of family medicine residents showed improvement in clinical skills at 6 weeks [19]. Observation moves beyond what a trainee knows, allowing assessment of what learners do in practice, a fundamental skill for taking care of patients. This objective assessment of cardiology fellows performing TTE reads moves beyond the standard reported numbers in a procedure log, a metric that has not been show in studies to represent technical or interpretive proficiency [4]. In our study, an expert (AQ) was able to offer concrete guidance on how novice trainees can cut down on wasteful practices such as remeasuring the PASP after you find the highest value in previewing the study or struggling with evaluating wall motion early in a study that has contrast imaging at the end. As a result direct observation of novices reading a TTE from start to finish should be considered when training fellows TTE interpretation.

In the radiology literature, teaching novices the scanning patterns of an expert prior to reading a chest X-ray consistently improved their diagnostic accuracy [11]. The same may hold for cardiology fellows and future studies should evaluate if the strategies and systematic processes used by experts, as highlighted in Table 2, improves fellow diagnostic accuracy and efficiency.

### Limitations

There are limitations to this study. This is a small, single center study and as such should be viewed as hypothesis generating. While we recruited 9 participants, similar numbers have been used in other CTA based studies and our findings allowed elaboration of institutional specific differences between novice, intermediate and expert readers [12, 20]. There may be additional factors that impact how studies are both taught and read at different institutions including institutional practice patterns, number of TTEs requiring interpretation each day, the complexity of cases, and the availability of sonographers to pre-read TTEs. Further studies should explore reading practices at other institutions and compare these to our findings (see Table 3).

**Table 3:** Primary diagnoses and coding elements scored for Cases 1–3

Case 1–21 Elements	Case 2–18 Elements	Case 3–9 Elements
Tricuspid Carcinoid*	Low gradient AS*	Tamponade Physiology*
Normal LV Size	Normal LV Size	Normal LV Function
Normal LV Function	*Decreased LV Function*	RVH Present
No Regional Wall Motion	Regional Wall Abnormalities Present**	Normal RV Function
Diastolic Dysfunction Present	LV Hypertrophy present	RVSP Severely Elevated**
Right Ventricle Hypertrophy (RVH) Present	Grade III diastolic dysfunction (i.e., restrictive)	Large Pericardial Effusion**
Normal Right Ventricle (RV) Function**	Normal RV Size	Any variation across mitral and tricuspid**
RV Septal Flattening Present	Mildly Reduced RV Function	IVC Distended without collapsibility**
Normal Left Atrial (LA) Size	Normal LA Size	No RV or RA Diastolic Collapse (as a result of elevated pulmonary pressures) **
Moderately Enlarged Right Atrial (RA) Size	Reduced Cardiac Output	
Aortic Regurgitation (AR) – Mild	Severe Aortic Valve Stenosis**	
Normal Aortic Valve Structure	Mild-Moderate MR	
Mild Mitral Regurgitation (MR)	Normal TR structure	
Mitral Valve Prolapse	Normal TR function	
Severe Tricuspid Regurgitation (TR)	RV Systolic Pressure (RVSP) > 50 mm Hg	
*Restricted Tricuspid Leaflets*	No Pericardial Effusion	
Hepatic Vein Reversal of Flow**	Normal Aortic Root	
Thickened Pulmonary Valve	IVC Distended without collapse	
Severe Pulmonic Regurgitation (PR) **		
No Pericardial Effusion		
IVC Size & RA pressure		
		Primary Diagnosis*
		Key Elements**

Bold highlights the primary diagnosis and the primary coding elements used to score Cases 1–3. Key elements which were felt to be important in obtaining a unified diagnosis are italicized. Each coded element (single diagnosis, 3 or 5 key elements and general coding elements) was designated as correct (1) or incorrect/missing (0) compared against a standardized report agreed upon by the adjudicating experts prior to study

Moreover, there are many factors (time, fund of knowledge, experience) that go into becoming an expert in any given skill not measured here. We understand that applying certain principles used by experts will not make novices experts but may allow novices to gain fundamental structural principles earlier in practice and learning to read more efficiently earlier in training. This study did not evaluate whether these changes help accelerate novice advancement. Future studies should consider when the interventions proposed should be introduced and if the development of these skills truly improves diagnostic accuracy or accelerates growth toward expertise. This study serves as a helpful starting point for reviewing educational practice patterns across institutions to create a framework for teaching cardiology fellows in training how to efficiently read echocardiograms.

## Conclusions

We identified important differences in the way experts and novices read echocardiograms which may inform how fellows could be training to improve their reading skills. These included (1) previewing the entire study before formally inputting the read, (2) initially viewing studies by multiple images to improve context clues for identification of structures (3) saving the interpretation of the hardest elements of the study for the end (4) filling out reports in discrete sections related to anatomy (left ventricle, right ventricle, valves and pericardium for example). In addition, all trainees found the use of direct observation to provide feedback on reading underutilized and informative. Although limited in generalizability the findings of this study suggests that a more systematic approach to TTE interpretation, using techniques found in experts, might be of significant value for trainees. Further study is needed to evaluate practice patterns at other institutions and assess whether implementation of these techniques by novices improves their diagnostic accuracy and efficiency of reading.

## Transcription Guidelines

There is NO patient identifying data in these recordings.

**Time-stamps 5 min**, **bold,** enclosed in brackets, and on its own line.

Template with line numbers on left-hand side.

Transcript doc should be named same as the audio file. Header should match audio file and doc. Please make sure transcription is not in tabular form.

## Speaker Identification:

- Interviewer I:Primary Speaker and Reader of Study
- Respondent R: Respondent (individual asking questions of primary reader)

## Echocardiography reading audio files

### Respondent typed verbatim/Interviewer verbatim

**Note:** It is important to capture a **more** accurate representation of the Interviewees actual speech, including long pauses and repeated words.

- **Time-stamp** every unheard portion or unheard word with **[inaudible 00:00:00]**
- **NOTE: only use inaudible** (not: too many voices, background noise, or audio glitch)
- Transcriber-asides for emphasis — [laughs], [coughs] etc.
- **[?]** use this behind every single instance in which we aren’t sure of a spelling or a name/term. Use it after every single use of the word, not only the first use of the word.
- Include side conversations, whether they seem relevant or not.
- Include ALL filler words such as “like, you know, I mean, so,” etc.
- Type slang as spoken.
- TYPE EVERYTHING.
- Include false starts and repeated words.
- Note long pauses with the use of ellipses **…**
- If someone cuts the speaker off, note it with the use of an em-dash **—**

**Abbreviations**

AI: Aortic insufficiency
AS: Aortic stenosis
ASD: Atrial septal defect
CO: Cardiac output
EF: Ejection function
HR: Heart rate
HTN: Hypertension
IVC: Inferior vena cava
LA: Left atria
LV: Left ventricle
LVOT: Left ventricular outflow tract
MR: Mitral valve
PA: Pulmonary artery
PASP: Pulmonary artery systolic pressure
PR: Pulmonic regurgitation
PS: Pulmonic stenosis
RA: Right atria
RV: Right ventricle
RVOT: Right ventricular outflow tract
TASPE: Tricuspid annular plane systolic excursion
TR: Tricuspid regurgitation
WMA: Wall motion abnormality
VTI: Velocity time integral

## Echo transcription glossary

### Medical terms commonly used

- Aortic valve (calcification)
- Atria.
- Carcinoid.
- Coaptation.
- Diastolic function.
- *E*/*A* ratio.
- Hypertrophy.
- Hypokinesis.
- Pleural Effusion.
- Substernal notch.
- Systolic function.
- Tamponade.
